# Use of Diagnostic Tests in Advanced Non–Small Cell Lung Cancer

**DOI:** 10.6004/jadpro.2017.8.2.5

**Published:** 2017-03-01

**Authors:** Beth Eaby-Sandy

**Affiliations:** Abramson Cancer Center, Hospital of the University of Pennsylvania, Philadelphia, Pennsylvania

## Abstract

**INTRODUCTION**

As the official publication of the Advanced Practitioner Society for Hematology and Oncology (APSHO), JADPRO is pleased to offer Part 1 of an accredited educational activity based on the recently concluded APSHO Regional Lecture Series. Hosted in collaboration with major cancer centers around the country, the APSHO Regional Lecture Series brought case-based didactic presentations and skills workshops to advanced practitioners.

In the spirit of JADPRO, three accredited Grand Rounds articles by Beth Eaby-Sandy, MSN, CRNP, OCN® (non–small cell lung cancer) and Sandra E. Kurtin, PhDc, ANP-C, AOCN® (multiple myeloma and chronic lymphocytic leukemia)—program chairs for the regional lecture series—offer the same practice-changing information and strategies for advanced practitioners.

In this Grand Rounds article, essentials skills of interpreting pathology reports and imaging studies are reviewed. Images from patient cases supplement the article.

Keep an eye out for Parts 2 and 3 in future issues of JADPRO, and be sure to check out apsho.org/lectures for information on registering for upcoming JADPRO Regional Lectures starting this spring.

**Use of Diagnostic Tests in Advances Non–Small Cell Lung Cancer**

This activity is supported by educational grants from AstraZeneca, Clovis Oncology, Lilly, and Merck & Co.

A continuing education article for nurse practitioners, clinical nurse specialists, advanced degree nurses, and oncology and hematology nurses.

**Release date:** March 13, 2017

**Expiration date:** November 13, 2017

**Expected time to complete activity:** 1.0 hour

**Meniscus Educational Institute**

3131 Princeton Pike,

Building 1, Suite 205A

Lawrenceville, NJ 08648

Voice: 609-246-5000

Fax: 609-449-7969

E-mail: lrubin@meniscusedu.com

**Journal of the Advanced Practitioner in Oncology**

94 N. Woodhull Road

Huntington, NY 11743

Voice: 631-692-0800

Fax: 631-692-0805

E-mail: claudine@harborsidepress.com

© *2017, Meniscus Educational Institute. All rights reserved.*

## Faculty

**Beth Eaby-Sandy, MSN, CRNP, OCN®**, Abramson Cancer Center, Hospital of the University of Pennsylvania, Philadelphia, Pennsylvania

## Activity Rationale and Purpose

Personalized medicine has become pervasive in oncology care today, requiring providers to first understand the science behind diagnosis, staging, and risk-adapted treatment selection, and then apply that knowledge to the ordering and interpretation of diagnostic testing and molecular testing for the management of various cancer diagnoses. Understanding of the molecular testing and radiology that is required during the initial staging as well as for follow-up to assess disease response to treatment, including the most appropriate tests and interpretation of test results (e.g. FISH, IHC, CT, PET/CT, and MRI) is also essential.

## Intended Audience

The activity’s target audience will consist of nurse practitioners, clinical nurse specialists, advanced degree nurses, and oncology and hematology nurses.

## Learning Objectives

After completing this educational activity, participants should be able to:

- Demonstrate a foundational understanding of how to interpret imaging studies that are used in the management of non–small cell lung cancer.

## Continuing Education

Statement of Credit—Participants who successfully complete this activity (including the submission of the post-test and evaluation form) will receive a statement of credit.

**Nurses**. This activity for 1.0 contact hour is provided by the Meniscus Educational Institute.

The Meniscus Educational Institute is accredited as a provider of continuing nursing education by the American Nurses Credentialing Center’s Commission on Accreditation.

Provider approved by the California Board of Registered Nursing, Provider No. 13164, for 1.0 contact hour.

## Financial Disclosures

All individuals in positions to control the content of this program (eg, planners, faculty, content reviewers) are expected to disclose all financial relationships with commercial interests that may have a direct bearing on the subject matter of this continuing education activity. Meniscus Educational Institute has identified and resolved all conflicts of interest in accordance with the MEI policies and procedures. Participants have the responsibility to assess the impact (if any) of the disclosed information on the educational value of the activity.

**Faculty**

**Beth Eaby-Sandy, MSN, CRNP, OCN®**, has acted as a consultant for Ariad; she has served on speakers bureaus for Amgen, Helsinn, and Merck.

**Lead Nurse Planner**

**Wendy J. Smith, ACNP, AOCN®**, has nothing to disclose.

**Content Reviewers**

**Anna Livers, ANP-BC, MSN, OCN®**, has nothing to disclose.

**Planners**

**Jeannine Coronna** has nothing to disclose.

**Claudine Kiffer** has nothing to disclose.

**Pamela Hallquist Viale, RN, MS, CNS, ANP**, has nothing to disclose.

**Lynn Rubin** has nothing to disclose.

**Annie Yueh** has nothing to disclose.

## Product Disclosure

This educational activity may contain discussion of published as well as investigational uses of agents that are not approved by the US Food and Drug Administration. For additional information about approved uses, including approved indications, contraindications, and warnings, please refer to the prescribing information for each product.

## How to Earn Credit

To access the learning assessment and evaluation form online, visit http://meded.hbrsd.com/

**Statement of Credit:** Participants who successfully complete this activity (including scoring of a minimum of 70% on the learning assessment and complete and submit the evaluation form with an E-mail address) will be able to download a statement of credit.

## Use of Diagnostic Tests in Advanced Non–Small Cell Lung Cancer

Interpreting pathology reports as well as routine imaging studies is one of the many roles of the advanced practitioner (AP) in oncology. With recent advancements in the management of patients with non–small cell lung cancer (NSCLC)—in particular, the advent of molecular targeted agents and immunotherapy—an understanding of molecular pathology reports and testing for immunotherapy markers is important in the management of NSCLC. These tests are performed differently and reported differently, but the results can drastically change first-line management for patients. In addition, the toxicity profiles and response patterns that appear on diagnostic imaging may vary. It is essential for APs to have a working knowledge of how to order, analyze, and apply these diagnostic tests.

## PATHOLOGY IN NSCLC 

**Case Study 1**

KB is a 31-year-old female current smoker who presents to the oncology clinic for the management of newly diagnosed metastatic NSCLC. Over the past 3 months, she has developed a persistent cough, which did not subside despite numerous treatments. A chest x-ray (CXR) revealed a left lower-lobe tumor. The tumor was biopsied via bronchoscopy and found to be a poorly differentiated carcinoma, which was strongly positive for cytokeratin 7 (CK7), carcinoembryonic antigen (CEA), and thyroid transcription factor-1 (TTF-1) but negative for CK20, estrogen receptor, and progesterone receptor.

**Figure 1:**
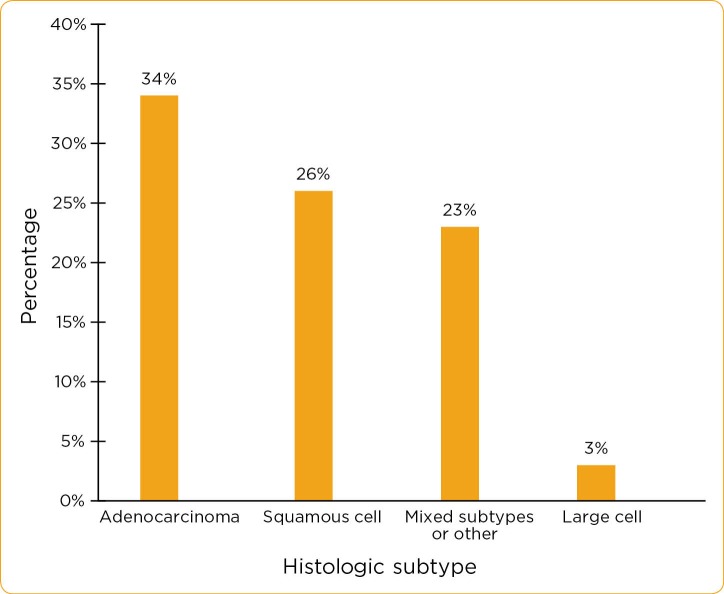
Histologic subtypes of non–small cell lung cancer: Percentage in men, US 2004–2009. Information from Houston et al. (2014).

The two most common histologic subtypes of NSCLC are adenocarcinoma and squamous cell carcinoma (Figure 1). Large cell makes up a small component of NSCLC; there are several mixed or "other" histologic variations (Houston, Henley, Li, White, & Richards, 2014). Adenocarcinoma is the most common histologic subtype in American men and women; although it is correlated with cigarette smoking, it is also the most common histologic subtype in nonsmokers with NSCLC (Underwood et al., 2012). Squamous cell carcinoma is most strongly associated with smoking cigarettes; therefore, these lesions tend to be centralized on imaging studies, where the smoke is most concentrated when inhaled.

When evaluating a pathology report for suspected lung cancer, it is important to understand the immunohistochemical (IHC) staining pattern applied by the pathologist to determine not only the origin of the tumor but also the histologic subtype. In the case of KB, she is a current smoker but also female and quite young. Therefore, it is necessary to rule out other origins, most importantly the possibility of breast cancer.

**Figure 2:**
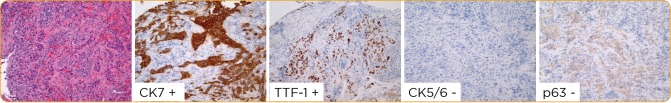
Pulmonary adenocarcinoma. CK = cytokeratin; TTF-1 = thyroid transcription factor-1. Images courtesy of Dr. Leslie Litzky, University of Pennsylvania.

**Figure 3:**
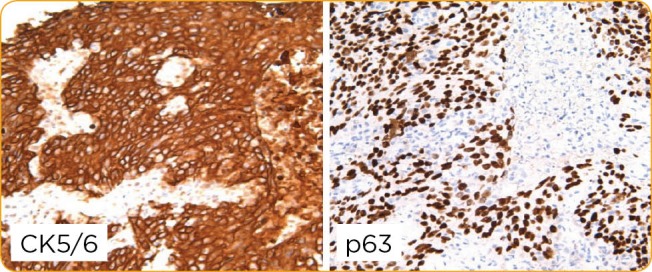
Squamous cell carcinoma. Typical immunohistochemical profile: cytokeratin (CK)5/6; p63 (crisp nuclear staining); calretinin-positive; thyroid transcription factor-1–negative. Images courtesy of Dr. Leslie Litzky, University of Pennsylvania.

Pathologists stain the cells for numerous markers to detect these differences to make a diagnosis. One of the most important stains is TTF- 1. When a tumor is positive for TTF-1, it is almost always adenocarcinoma arising from the thyroid or lung. See the Table below for a list of commonly used stains in NSCLC and their clinical applications and Figures 2 and 3 for a look at how these stains alter the view of the cells.

**Table 1:**
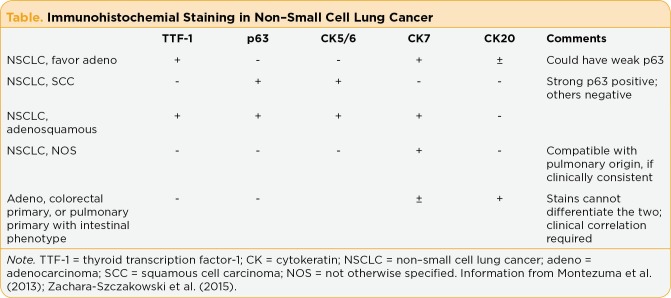
Immunohistochemial Staining in Non–Small Cell Lung Cancer

KB’s tumor tests negative for estrogen receptor (ER) and progesterone receptor (PR), decreasing the likelihood of breast cancer. Given her smoking history, imaging characteristics, and the TTF-1 and cytokeratin 7 (CK7) positivity of her staining pattern, the diagnosis is consistent with an adenocarcinoma of pulmonary origin. Since she has a nonsquamous NSCLC, she should be tested, at minimum, for epidermal growth factor receptor (EGFR), anaplastic leukemia kinase (ALK), ROS proto-oncogene 1 (ROS1), and programmed cell death ligand 1 (PD-L1; National Comprehensive Cancer Network [NCCN], 2016).

## MOLECULAR PATHOLOGY IN NSCLC

It is feasible to test most NSCLC samples for molecular alterations or to conduct PD-L1 testing to determine the efficacy of particular immunotherapy drugs. As APs, we rely on adequate tissue samples to provide us this information. Even among experienced pathologists, there can be disagreement about the interpretation of histology (Grilley-Olsen et al., 2009); thus, the largest specimen that can be safely obtained will often offer optimal testing. Some caveats are small percutaneous biopsies that do not yield sufficient cellularity and bone biopsies that may be decalcified for histologic interpretation, which often renders this tissue unavailable for molecular testing (Vanderlaan et al., 2014).

As noted in Case Study 1, it is important to test for EGFR, ROS1, ALK, and PD-L1 in all patients with NSCLC who have a nonsquamous histology. Testing patients of squamous histology who may fit the clinical profile of having one of these mutations, such as a female never-smoker of Asian ethnicity, could also be considered (Ha et al., 2015). In addition, testing as part of a larger panel of mutations and genetic alterations is encouraged when possible, as it could lead to detecting other rare mutations, which may give the patient the option of participating in a clinical trial.

Another option is a blood test to detect these mutations and alterations, often referred to as a "liquid biopsy." There are several companies that perform these blood tests, along with several advantages and disadvantages of this type of testing. The obvious advantage is that it can be done without an invasive procedure and usually requires only about two tubes of blood obtained during routine phlebotomy. This option is particularly attractive when looking for the *EGFR* T790M mutation (an acquired resistance mutation to EGFR) if a patient’s disease is progressing on EGFR therapy. However, liquid biopsy relies on DNA shedding from the tumor into the bloodstream, which can vary. As a result, the sensitivity of liquid biopsies may vary widely (Pérez-Callejo, Romero, Provencio, & Torrente, 2016).

Two meta-analyses that looked at the sensitivity and specificity of *EGFR* detection on liquid biopsy revealed a sensitivity of 67.4% (95% confidence interval [CI]: 51.7%–80%) and a specificity of 93.5% (95% CI: 88.8%–96.3%), and a sensitivity of 62%( 95% CI: 51.3%–71.6%) and a specificity of 95.9% (95% CI: 92.9%–97.7%), respectively (Luo, Shen, & Zheng, 2014; Qiu et al., 2015). When evaluating for the *EGFR* T790M mutation, one study found that the sensitivity for detection in plasma was 70% in patients with a confirmed tissue diagnosis of *EGFR* T790M (Oxnard et al., 2016). Interestingly, of patients whose tissue tested negative for *EGFR* T790M, the blood test detected a mutation in around 30% of them.

These results highlight the fact that tumors are heterogeneous, and thus results may be missed on tissue biopsy; vice versa, if there is insufficient DNA shedding into the plasma, liquid biopsy may be falsely negative as well. Therefore, it is reasonable to start with the least invasive test of liquid biopsy. However, if the results are negative, one would proceed with a procedure for biopsy.

**Case Study 2**

**Figure 4:**
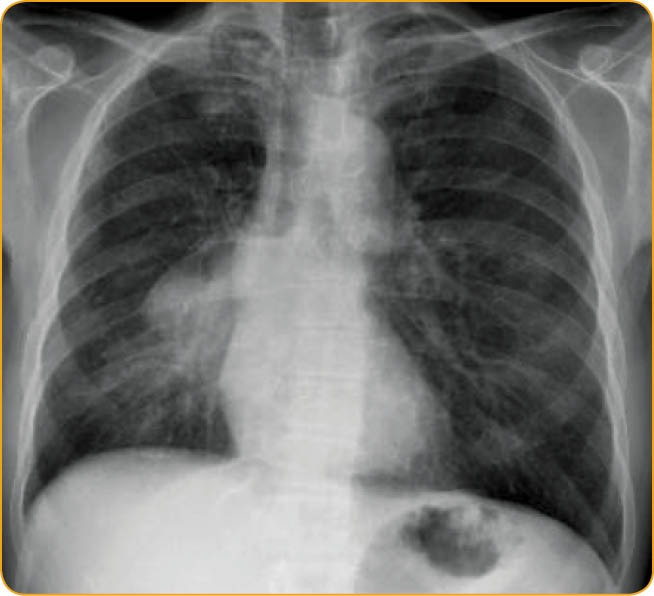
Chest x-ray of patient in Case Study 2.

JO is a 58-year-old male working per diem jobs without insurance who rarely goes to the doctor. He is a pack-a-day smoker. He developed a cold with a wheeze that did not improve with over-the-counter medications. He presented to the emergency department, and his CXR is shown in Figure 4.

## RADIOLOGIC INTERPRETATION

**Figure 5:**
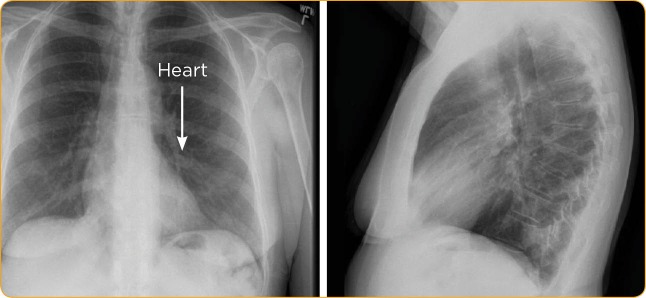
Posteroanterior (left) and lateral (always left-sided; right) views of chest x-ray of patient in Case Study 2.

A chest x-ray is often the first, easiest, and most inexpensive diagnostic test used in NSCLC for evaluating for any abnormality, whether it is a tumor, pneumonia, pleural effusion, or something else. JO was found to have a right lower-lobe mass. Normal CXRs, as seen in Figure 5, will reveal open airspaces, a normal-sized heart overlapping part of the left lower lobe of the lung, a normal (not widened) mediastinum, and both of the costophrenic angles sharp (coming to a point). There should be a posteroanterior (PA) view and a lateral view, always shot from the left side, with the patient facing the machine. The patient should be at full inspiration, with arms above the head on the lateral view.

**Figure 6:**
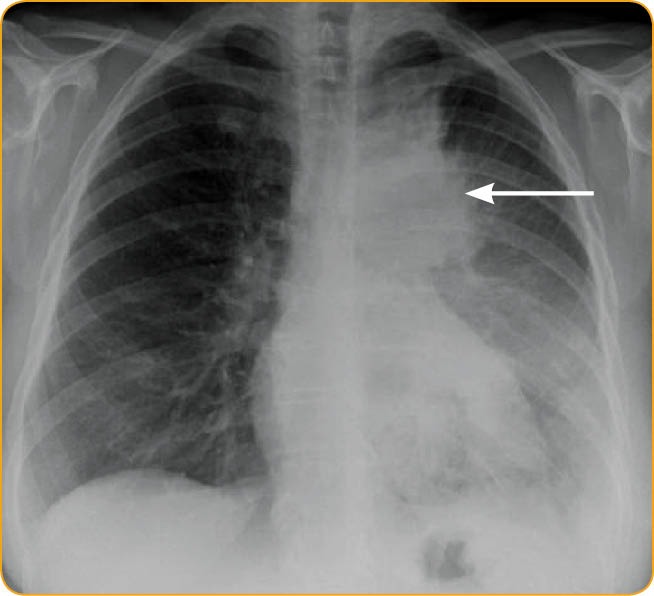
Chest x-ray shows a mass in the left upper lobe (arrow).

**Figure 7:**
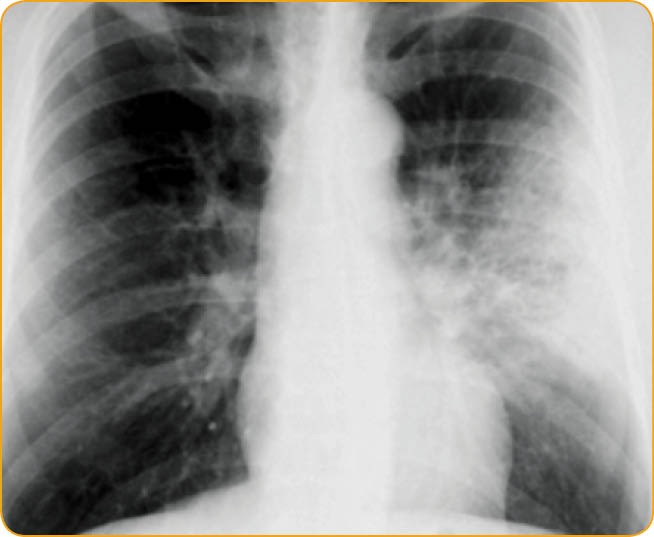
Chest x-ray shows pneumonia.

**Figure 8:**
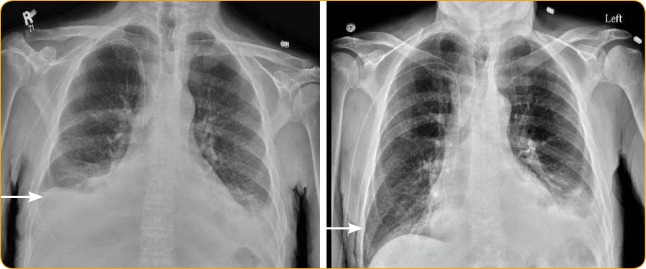
Chest x-rays show right-sided pleural effusion (left) and the same patient 1 day after drainage (right).

A CXR can show obvious changes but may not detect very small abnormalities. Figure 6 is an example of a large left upper-lobe mass. Note the solid, round component with some corresponding atelectasis in the left lower lobe. Conversely, in Figure 7, this is more likely an airspace disease such as a pneumonia; note the patchy consolidative appearance. Figure 8 shows a patient with a bilateral pleural effusion, with the right side greater than the left side. Note the look of the right costophrenic angle; it is blunted with fluid prior to drainage and then sharply seen after fluid has been drained.

**Case Study 2 Continued**

JO undergoes a computed tomography (CT) of the chest, which reveals a large right lower-lobe mass that is causing narrowing of his airways. He also has a right hilar lymph node, enlarged mediastinal lymph nodes, and a thickened adrenal gland. Positron-emission tomography (PET)/CT confirms the avidity of these lesions from the fluorodeoxyglucose (FDG) dye.

A CT scan is the most commonly used test for diagnosis, surveillance, and evaluation of disease response to treatment of NSCLC. Clinicians usually look at the horizontal CT images. There are also several views, such as the lung windows, chest/abdomen windows, and bone windows, which help to show different structures in different ways.

**Figure 9:**
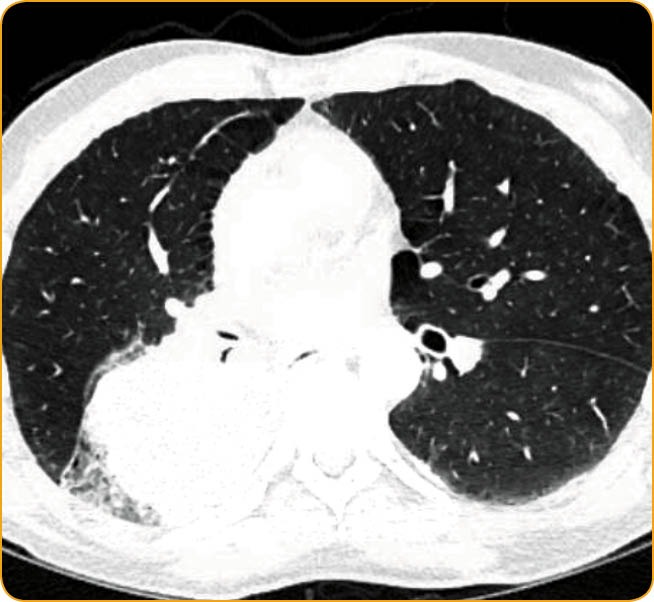
CT lung window shows the lung parenchyma

The lung windows, an example of which is shown in JO’s scan in Figure 9, show the lung parenchyma, which is most helpful to see a primary tumor or any tumor within the lung tissue itself. Most of the tiny white spots that canvas both lungs are the microvasculature of the lungs. Also seen in Figure 9 is how the large tumor is pinching off the larger right lower-lobe airways, putting the patient at risk for shortness of breath and pneumonia.

**Figure 10:**
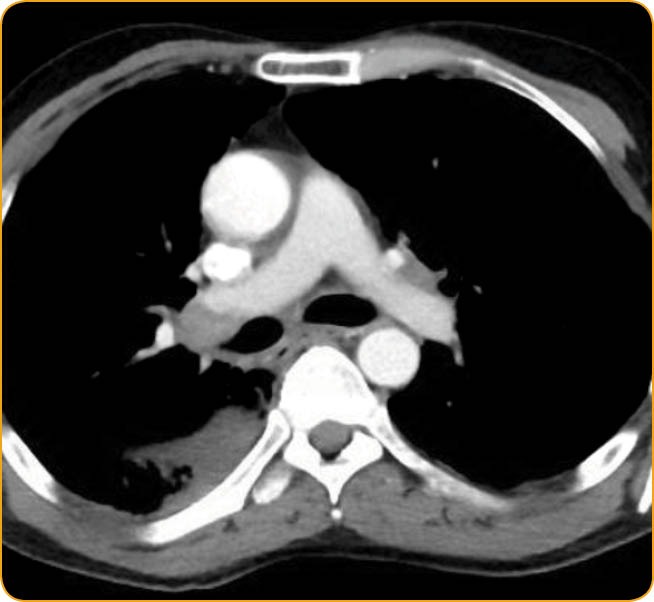
CT chest/abdomen window shows the enlarged right hilar lymph node.

**Figure 11:**
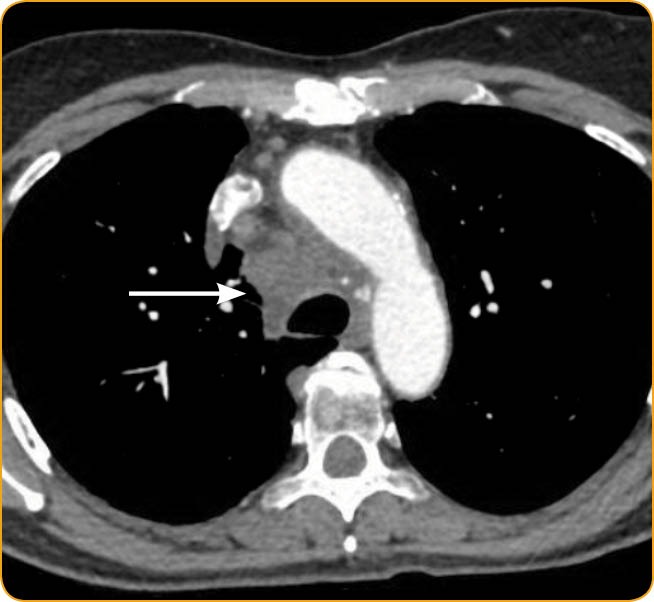
CT scan shows a large, 2.5-cm malignant mediastinal lymph node (arrow).

**Figure 12:**
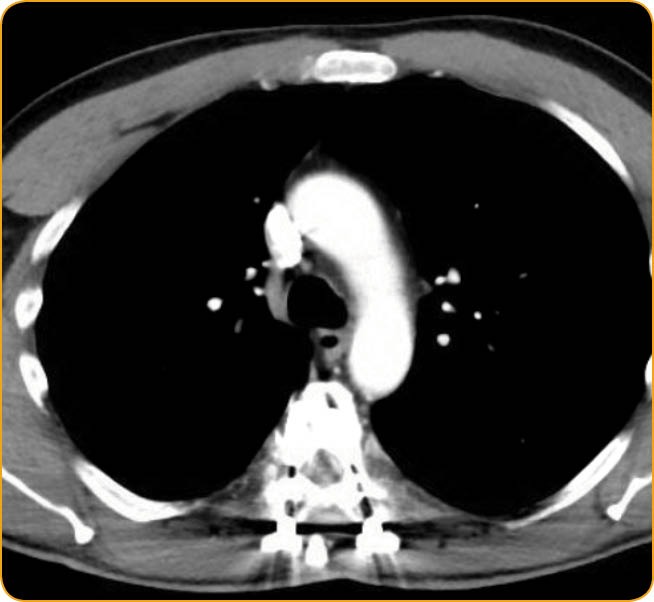
CT scan shows a normal-looking mediastinum.

The chest/abdomen windows are most helpful to evaluate for lymphadenopathy, especially when IV contrast has been administered properly. In Figure 10, there is an enlarged right hilar lymph node, and in Figure 11, there is an enlarged mediastinal lymph node. Note that Figure 12 shows how a normal mediastinum should look in a healthy individual.

**Figure 13:**
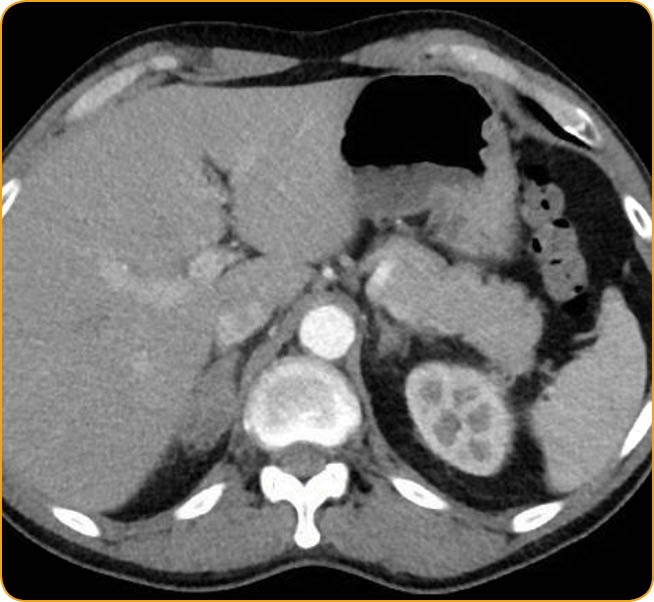
CT image shows the liver, which appears to be clear, but the adrenal gland is thickened and enlarged on the right side.

Also, the chest-abdomen windows are best to view the liver and structures in the upper abdomen on a CT chest scan. In Figure 13, the liver appears to be clear, but the adrenal gland on the right side is thickened and enlarged. If this patient could not receive IV contrast, the liver would appear homogenous and would be difficult to evaluate for metastases, hence the importance of ordering CT scans with contrast.

**Figure 14:**
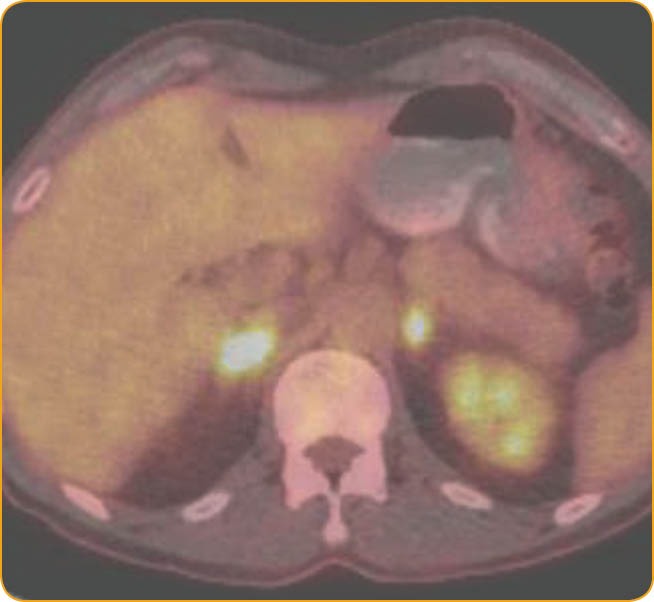
PET fusion image shows the thickened right adrenal gland from the CT scan of the patient in Case Study 2 and the intensity of the FDG dye in the left adrenal gland.

Positron-emission tomography is commonly used in combination with CT scanning as a fusion study to evaluate abnormalities found on the CT scan to see whether they uptake FDG. The scan is usually ordered from the skull base to the mid-thigh, except for patients with melanoma, where a whole-body PET/CT is indicated. The FDG uptake is reported on a scale by a radiologist; the higher the number is, the more suspicious the lesion is for cancer. For example, Figure 14 shows the thickened right adrenal gland from JO’s CT scan. Of note is the intensity of the FDG dye in the left adrenal gland, which was not noted to be abnormal on the CT scan. This highlights the importance of PET/CT imaging in detecting sometimes inconspicuous findings from routine CT imaging.

**Case Study 1 Continued**

**Figure 15:**
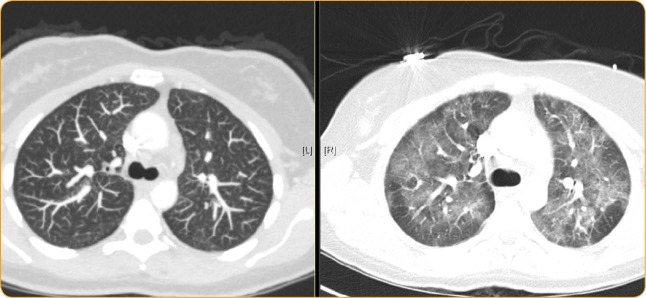
The CT scan on the left was taken prior to starting treatment with crizotinib. The CT scan on the right is an example of pneumonitis occurring 3 weeks after starting crizotinib.

Our first patient KB is found to have an *ALK* gene translocation in her tumor. She begins therapy with crizotinib (Xalkori), an oral tyrosine kinase inhibitor that targets *ALK*. After about 3 weeks from starting crizotinib, she calls the nurse practitioner and reports a new onset of significant shortness of breath and cough without mucus. She comes into the office and has a CT chest done to evaluate the potential cause. Figure 15 shows the CT scan prior to her starting treatment with crizotinib (at left) and the CT scan done in the office 3 weeks after starting the drug (at right).

## COMMON CT ABNORMALITIES IN NSCLC

Pneumonitis, also sometimes called interstitial lung disease (ILD), is an inflammation of the lung tissue often caused by drug toxicity. The scan on the right of Figure 15 is an example of pneumonitis occurring 3 weeks after starting crizotinib. The bilateral effect on the lungs indicates a systemic process. Furthermore, the cloud-like, patchy, and widely disseminated appearance without consolidation indicates drug toxicity rather than diffuse, bilateral pneumonia.

**Figure 16:**
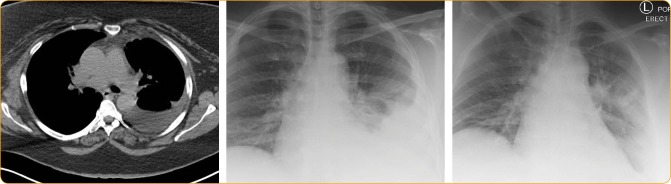
CT scan of the chest (left) shows pleural effusion. Chest x-rays done on the same day as the CT scan, predrainage (middle) and postdrainage (right).

Pleural effusion is also a common occurrence in patients with NSCLC. Figure 16 shows a left-sided CT image of a pleural effusion. It can be noted that the appearance of the CT image of the effusion differs from the way it looks on the CXR views.

**Figure 17:**
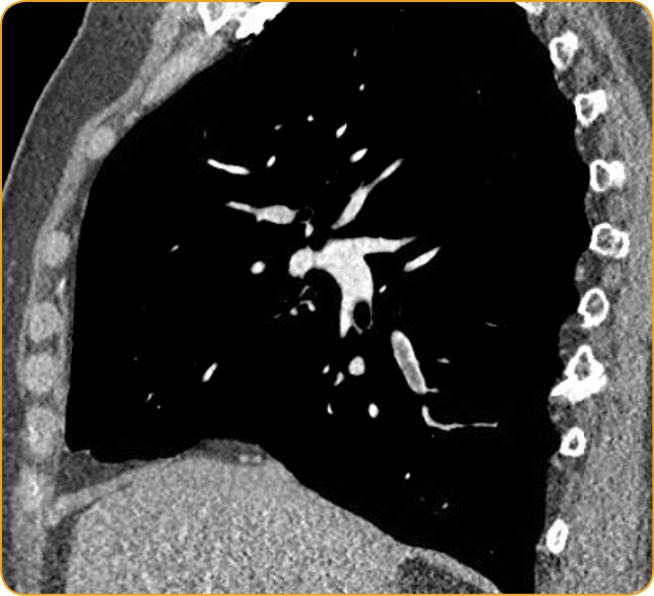
CT scan shows pulmonary embolism.

**Figure 18:**
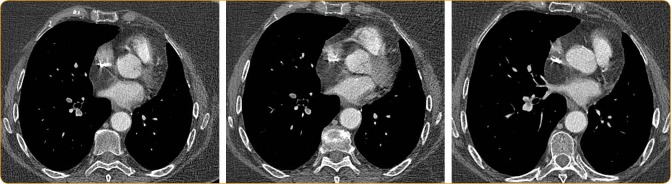
Pulmonary embolism as seen in the vessels in the horizontal slides of the CT scan.

Pulmonary embolism (PE) is another common complication in patients with NSCLC. For the untrained eye, it can be very difficult to find. Most often, APs will need to rely on the radiologist to make this call. It is best seen on a CT scan with IV contrast, when the scan is specifically ordered as a PE protocol, where the technician will time the contrast precisely and perform numerous, very thin slices of vasculature. Figure 17 is a sagittal image from a CT scan showing the filling defect of a vessel indicating a PE. Figure 18 illustrates PE as seen in vessels in the horizontal slides of a CT scan. When PE is suspected, it’s recommended that APs evaluate images and call the radiologist to confirm the existence or absence of PE.

**Figure 19:**
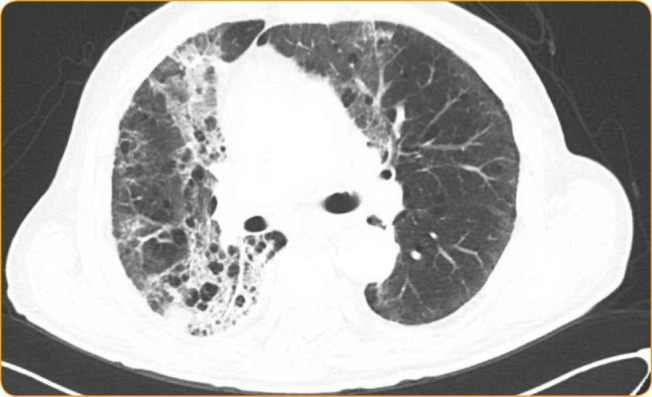
CT scan shows a large part of the radiated right lung and the mediastinum radiation.

Radiation pneumonitis is an inflammation of lung tissue occurring after a period following the completion of radiation therapy. It is a reaction to the radiation and follows in the same field as the radiation was administered. Figure 19 shows a patient who had a large part of the right lung radiated and the mediastinum radiation. The linear appearance in the right lung can be seen, with most of the lung clear, and only the small area within the radiation field of the right lung affected.

## CONCLUSIONS

Advanced practitioners are often required to order diagnostic tests, including either diagnostic or liquid biopsies, molecular testing on pathology specimens, and a variety of other diagnostic imaging tests. Different biopsy methods carry their respective risks; it is important for APs to be able to explain to patients the risks and benefits of the different biopsy techniques and the yield for molecular testing. In addition, APs must also be able to explain to their patients the reasons for ordering biopsy and molecular testing procedures.

An understanding of proper ordering procedures for diagnostic imaging is also required. A knowledge of the way the contrast is ordered, the proper protocols to follow, and other variables will ensure patients receive the correct test that yields clinically appropriate results. Practicing reading CXRs, CT scans, and PET/CT scans can help APs become familiar with the anatomy and abnormalities as they are presented. As oncology becomes a more specialized field, with more targeted treatments with different toxicity profiles, oncology APs must stay educated on proper diagnostic testing.

**Disclosure**

Ms. Eaby-Sandy has acted as a consultant for Ariad; she has served on speakers bureaus for Amgen, Helsinn, and Merck.

**How to Earn Credit**

To access the learning assessment and evaluation form online, visit http://meded.hbrsd.com/

**Statement of Credit:** Participants who successfully complete this activity (including scoring of a minimum of 70% on the learning assessment and complete and submit the evaluation form with an E-mail address) will be able to download a statement of credit.
